# Encapsulation Techniques of Carotenoids and Their Multifunctional Applications in Food and Health: An Overview

**DOI:** 10.1002/fsn3.70310

**Published:** 2025-05-20

**Authors:** Muhammad Tayyab Arshad, Sammra Maqsood, Ali Ikram, Ammar Ahmad Khan, Awais Raza, Aneeq Ahmad, Kodjo Théodore Gnedeka

**Affiliations:** ^1^ University Institute of Food Science and Technology, The University of Lahore Lahore Pakistan; ^2^ National Institute of Food Science and Technology, University of Agriculture Faisalabad Faisalabad Pakistan; ^3^ University Institute of Diet and Nutritional Sciences, The University of Lahore Lahore Pakistan; ^4^ Togo Laboratory: Applied Agricultural Economics Research Team (ERE2A) University of Lomé Lome Togo

**Keywords:** freeze drying, pigments, spray drying, β‐Carotene

## Abstract

Carotenoids are a broad category of biologically active pigments in plants and animals with significant health associations, including immune‐modulatory, anti‐inflammatory, and antioxidant roles. They persuade prevention of illness through numerous mechanisms such as protection against oxidative stress, encouragement of cardiovascular and neuroprotective events, and reduction of diseases comprising cancer and macular degeneration. Carotenoids have several health benefits but are unstable, with low bioavailability, and easily degrade in environments containing light, heat, and oxygen. Since the encapsulation improves carotenoid solubility, stability, and controlled release, it has become a feasible strategy for overcoming these issues. This review considers a few encapsulation methods, including electrospinning, lipid‐based delivery systems, spray drying, freeze drying, and supercritical fluid technology. Each of these methods is assessed in terms of their ability to retain carotenoids, improve bioavailability, and deliver targeted distribution. Evaluation of these methods has been made with the view of benefits, drawbacks, and suitability for industrial use. In conclusion, the analysis identifies current challenges in carotenoid encapsulation and potential future investigation and innovation directions in this area to exploit carotenoid‐based functional foods, nutraceuticals, and medications.

## Graphical abstract

This review discusses various encapsulation strategies, including electrospinning, lipid‐based delivery systems, spray drying, freeze drying, and supercritical fluid technology. Each technique is critically assessed regarding its potential to improve carotenoid retention and bioaccessibility while considering its industrial feasibility. By synthesizing current advancements and identifying key challenges, our study offers valuable insights into future research directions to optimize carotenoid‐based functional food formulations and pharmaceuticals.

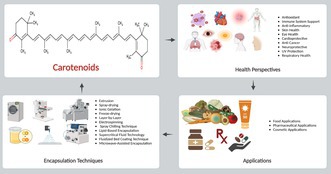

## 1 Introduction

Carotenoids, formed by bacteria, fungus, algae, and plants, represent a diverse group of certainly occurring pigments; these are intricate both in photosynthesis and in photoprotection (Maoka 2020). Such composites contribute the red, orange, and yellow colors to fruits and vegetables, such as bell peppers, tomatoes, and carrots (Ikram et al. 2024; Adadi et al. 2018). Rendering to their chemical composition, carotenoids are broadly divided into two groups: xanthophylls, such as lutein, zeaxanthin, and astaxanthin, and carotenes, such as lycopene and β‐carotene (Tufail et al. 2024; Bhatt and Patel 2020). Xanthophylls contain oxygen functional groups, making them more soluble in aqueous conditions. On the other hand, carotenes are hydrocarbon compounds only (Martins and Ferreira 2017). Because humans do not have the biochemical tackle to create them, carotenoids must be gotten from the diet (Eggersdorfer and Wyss 2018). Food matrix, processing, and interface with dietary lipids are some of the factors that disturb the bioavailability of carotenoids (Moran et al. 2018). Carotenoid pigments have been proven to be additional bioavailable when cooked or processed in a way those disruptions down plant cell walls, releasing them from the matrix (Rodriguez‐Concepcion et al. 2018). Sources of microalgae, such as *Haematococcus pluvialis* and *Dunaliella salina* , which are rich in β‐carotene and astaxanthin, can also be used to extract carotenoids (Hamed et al. 2023). One of the present carotenoid production approaches that aid in reducing dependency on traditional farming practices is sustainable microbial fermentation (Fernandes et al. 2018). Carotenoids help to decrease the incidence of chronic diseases such as cancer, heart disease, and neurological disorders by shielding cells from oxidative stress, as explained by Eggersdorfer and Wyss (2018). The cell acts to avert this kind of oxidative damage to DNA, lipids, and proteins as reactive oxygen species can cause them (Saini, Prasad, et al. 2022; Saini, Ranjit, et al. 2022). As stated by Rodriguez‐Concepcion et al. (2018), lycopene, contained mainly in watermelon and tomatoes, can minimize the risk of atherosclerosis and LDL cholesterol, thus indicating cardioprotection. However, according to Zia‐Ul‐Haq et al. (2021), β‐carotene serves as the key precursor for vitamin A, which is indispensable for the proper working of the immune system, normal vision, and healthy skin. Retinas store carotenoids such as lutein and zeaxanthin. These are some of the vital components of the eyes because they save the eyes from the destructive blue light that speeds up the progression of age‐related macular degeneration (Moran et al. 2018). In population studies, better levels of lutein and zeaxanthin are associated with better visual functions with a reduced rate of cataract incidences (Maoka 2020). Carotenoids have anti‐inflammatory and antiproliferative effects, which can stop the proliferation of cancer by redirecting communication pathways and pretumor onset (Bhatt and Patel 2020). It has also been reported that people who consume higher levels of carotenoids are least likely to develop lung, breast, and prostate cancers. Though, the benefits may vary depending on person to person with variables such as genetic makeup and personal lifestyle issues (Focsan et al. 2019). Another benefit of carotenoids discovered is neuroprotective. Recently, an investigation discovered that astaxanthin upsurges cognitive functions while avoiding the incidence of Alzheimer's disease (Rehman et al. 2020). Although carotenoids have many health benefits, they are less valuable in medicinal and culinary submissions because of their low constancy and bioavailability (Fernandes et al. 2018). These compounds have to be safeguarded from oxidative breakdown in the presence of heat, light, and oxygen to ensure their activities (Boonlao et al. 2022). The addition of a matrix in the encapsulation process improves the solubility and stability of carotenoids by protecting them from environmental restrictions (Chen et al. 2017). Multifaceted coacervates, polymeric nanoparticles, liposomes, and nanoemulsions are the encapsulating techniques that are studied; each one has its merits concerning the cosmetic delivery of carotenoids (Focsan et al. 2019). One of the significant reasons why nanoencapsulation is crucial is because it has the potential to upsurge bioavailability and cellular uptake due to its improved dispersibility in water (Rehman et al. 2020). As indicated by Hamed et al. (2023), encapsulated carotenoids exhibit a better antioxidant capacity and shelf life than their free counterparts since they are absorbed better in the gastrointestinal tract. Because emulsions ensure greater bioaccessibility and retarded release characteristics, there has been much research done on the emulsion‐based delivery systems for both β‐carotene and lycopene (Boonlao et al. 2022). In addition, encapsulated microalgal carotenoids have positively been incorporated into functional foods and nutraceuticals, which has greatly expanded their applications in the health and wellbeing sector (Hamed et al. 2023). Carotenoids are essential bioactive composites with many health benefits, but their low stability and bioavailability sometimes limit their efficiency. Encapsulation techniques have become a real‐world means of refining carotenoid absorption, protection, and targeted administration. To improve the availability of carotenoids in functional foods, pharmaceuticals, and nutraceuticals, future investigation should emphasize refining encapsulation strategies to attain and commercialize large‐scale production.

The purpose of this review is to comprehensively investigate the health benefits of carotenoids and highlight how progressive encapsulation techniques can improve their stability and bioavailability. Carotenoids are broadly renowned for their immunoenhancing, anti‐inflammatory, and antioxidant properties. They play a significant role in preventing many chronic diseases, such as cancer, neurological diseases, and cardiovascular disorders. However, barriers to their effective application are their low bioavailability, low solubility, and susceptibility to degradation. This review article attempts to objectively assess which encapsulation methods, such as spray drying, freeze drying, lipid‐based carriers, and supercritical fluid technology, better advance carotenoid delivery and protection. A contrast of these methods helps in selecting the most encouraging method for optimizing carotenoid encapsulation rummage‐sale in the food, pharmaceutical, and cosmetic industries.

## 2 Carotenoids: Health Benefits and Mechanisms

**FIGURE 1:**
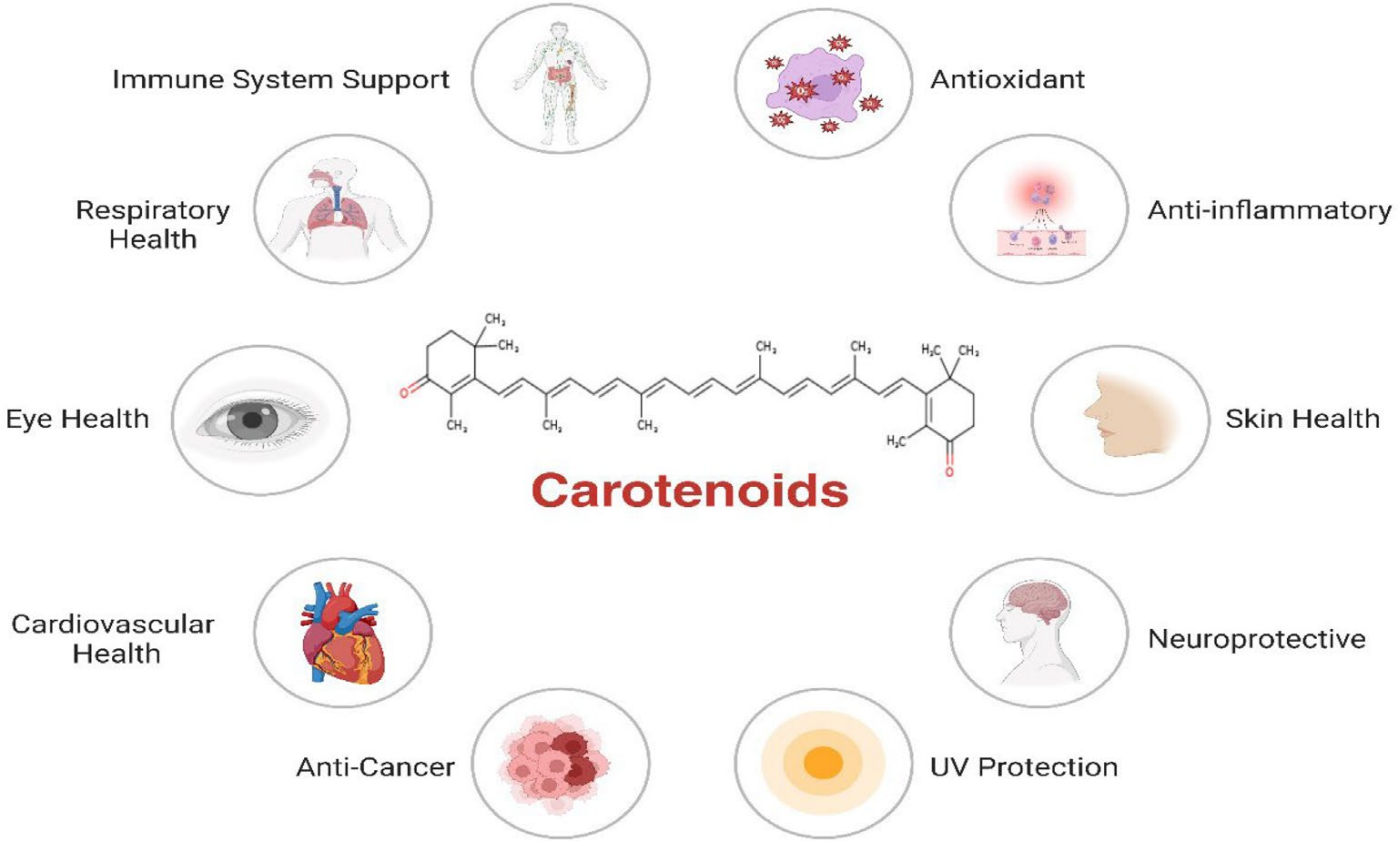
Health potential of carotenoids.

Carotenoids are well known for their health benefits: a broad class of pigments arising naturally in plants, algae, and photosynthetic bacteria (Figure 1). The substances have anti‐inflammatory, immune‐modulating, and antioxidant properties, and their potential to avoid and treat chronic illnesses is being increasingly explored. Their exceptional advantages and mechanisms are included below:

### 2.1 Antioxidant Activity

Carotenoids, which are powerful antioxidants, can neutralize ROS, a leading reason for cellular damage and chronic diseases, plummeting oxidative stress. The conjugated double‐bond structure of carotenoids confers the ability to scavenge free radicals and quench singlet oxygen, thus bestowing antioxidant properties on them (Young and Lowe 2018; Ribeiro et al. 2018). For instance, there is the example of β‐carotene, which completely scavenges singlet oxygen and inhibits lipid peroxidation in the cell membrane (Merhan 2017). Similarly, the human retina's abundant lutein and zeaxanthin are crucial for averting age‐related macular degeneration (Bungau et al. 2019). Carotenoids donate electrons to alleviate ROS and transform them into less reactive species, through which they achieve their antioxidant actions. According to one study by Squillaci et al. (2017), carotenoids that are formed from *extremophilic* microorganisms hold great potential as antioxidants with the capability to prevent in vitro oxidative damage. In addition, the intake of carotenoids as supplements decreases oxidative stress indicators (Eggersdorfer and Wyss 2018).

### 2.2 Immune System Support

By regulating the immune cell activity and cytokine amalgamation, carotenoids help endorse immunological functions. According to Nabi et al. (2020), provitamin a carotenoids, such as β‐carotene, as well as nonprovitamin a carotenoids, encourage and improve immune responses. Retinoids, controlling the expansion and proliferation of T cells and B cells, the two important constituents of adaptive immunity, are generated by the metabolism of β‐carotene (Toti et al. 2018). Carotenoids impact immune function by acting through nuclear receptors such as retinoic acid receptors (RARs) and retinoid X receptors (RXRs). This interaction has been shown to express genes related to immune defense more frequently. For example, Tan et al. (2020) revealed that dietary carotenoids increased the immune response in invertebrates by promoting the activity of antimicrobial peptides. Similarly, the clinical study by Khadim and Al‐Fartusie (2021) on carotenoid supplementation found the improvement of immunological parameters among patients with impaired immune systems.

### 2.3 Anti‐Inflammatory Effects

Anti‐inflammatory activity of carotenoids is due to their ability to inhibit proinflammatory cytokines and pathways, such as cyclooxygenase‐2 (COX‐2) and nuclear factor kappa B (NF‐κB) (Milani et al. 2017; Hajizadeh‐Sharafabad et al. 2022). Carotenoids prevent disease conditions such as cardiovascular diseases, neurological disorders, and nonalcoholic fatty liver disease (NAFLD) by reducing inflammation. Carotenoids scavenge ROS that initiate inflammatory pathways, thus resulting in decreased inflammation. Such is the case regarding lycopene and β‐carotene whereby they have demonstrated the ability to reduce inflammation by decreasing TNF‐α and IL‐6, and also downregulating NF‐κB signaling of proinflammatory cytokines (Kawata et al. 2018). A systematic review of animal studies by Silva Meneguelli et al. (2024) highlighted improved gut health and reduced systemic inflammation caused by carotenoid supplementation that alters gut microbiota and epithelial barrier integrity. In vitro demonstration of the anti‐inflammatory and anticancer potential of microalgal carotenoids was done by Ávila‐Román et al. (2021) by highlighting their probable therapeutic applications. Due to their anti‐inflammatory, immunoprotective, and antioxidant properties, carotenoids can deliver great health benefits. Their functional mechanisms include scavenging ROS, regulating immunological activities, and inhibiting inflammatory pathways. Case studies and clinical data have shown how carotenoids may advance immunity, reduce inflammation, and handle diseases with a common basis in oxidative stress. These significances highlight the importance of including those foods or supplements rich in carotenoids in the diet for improved health concerns.

### 2.4 Skin Health and Protection

Carotenoids such as lutein, β‐carotene, and colorless phytoene and phytofluene are essential for skin health because of their antioxidant and photoprotective qualities. They accumulate in the epidermis and dermis of the skin to counteract ROS induced by UV radiation, thus preventing oxidative damage and lipid peroxidation (Darvin et al. 2022; Zerres and Stahl 2020). Meléndez‐Martínez et al. (2019) have also pointed out that phytoene and phytofluene are able to absorb UV light and significantly diminish inflammation and DNA damage triggered by UV radiation. Carotenoids present in the dietary supplement improve skin quality; they render the skin soft and pliable, hydrating, protecting against UV rays, robust, and reddening‐prone (Baswan et al. 2021; Zerres and Stahl 2020). Such findings are proven through case studies and clinical trials that indicate the importance of surgical carotenoids for the care of the skin and the relief of environmental stress. For instance, Balić and Mokos (2019) suggested the significance of carotenoids in dermatological health by consuming food or dietary supplements rich in carotenoids can significantly prevent oxidative damage to the skin to give the view of beautiful healthy skin.

### 2.5 Eye Health (Reducing Macular Degeneration Risk)

Two well‐known carotenoids, which have been associated with eye health and a decreased risk of AMD, are lutein and zeaxanthin. The carotenoids selectively segregate within the macula, an area of the retina, to act as antioxidants and light screeners to combat inflammation and oxidative stress (Mrowicka et al. 2022; Lem et al. 2021). It has been found that the supplementation or intake of diet with lutein and zeaxanthin has relatively less impact on the incidence of AMD. According to Kim, Choi, et al. (2018) and Kim, Kim, et al. (2018) older women who consumed fruits and vegetables with high levels of carotenoids have a significantly lower frequency of AMD. Similarly, Jiang et al. (2023) reported that improved macular health was associated with higher dietary carotenoid intake among Chinese populations. These carotenoids molecular functions in shielding retinal cells from oxidative impairment were further revealed by Johra et al. (2020) to highlight their significance in the prevention of AMD and in sustaining general visual function.

### 2.6 Cardiovascular Health

By reducing inflammation and oxidative stress, main contributors to CVD, carotenoids such as lycopene and β‐carotene greatly increase cardiovascular health. As they improve lipid profiles, these substances avert oxidation of LDL and are a primary causative factor in the development of atherosclerosis (Bohn et al. 2021; Kulczyński et al. 2017). For example, lycopene has been shown to reduce blood pressure, ease arterial stiffness, and advance endothelial function; such effects result in a decline in the risk of heart disease and stroke (Gammone et al. 2017). Such positive impacts are supplemented by some clinical trials that demonstrated the relationship between a higher level of carotenoids in the bloodstream and a low risk of CVD (Eggersdorfer and Wyss 2018). The anti‐inflammatory properties of carotenoids also modify inflammatory pathways, providing additional protection in contradiction to cardiovascular damage. For illustration, an investigation reviewed by Kulczyński et al. (2017) acmes the potential of carotenoid‐rich diets in the deterrence and control of CVD and highlights their participation in improving heart health pointers (Table 1).

**TABLE 1:** Health benefits of carotenoid encapsulation.

Health benefit	Key carotenoids	Mechanism	Encapsulation benefits	Evidence	References
Antioxidant	β‐Carotene, lycopene	Scavenges free radicals	Enhances stability	Reduces oxidative markers	Bohn et al. (2021)
Immune support	Astaxanthin	Boosts T‐cell function	Improves bioavailability	Enhances immunity	Tan et al. (2020)
Anti‐inflammatory	Lycopene	Inhibits NF‐κB pathway	Controlled release	Reduces CRP levels	Milani et al. (2017)
Skin health	Astaxanthin	UV protection	Prevents degradation	Improves elasticity	Darvin et al. (2022)
Eye health	Lutein, Zeaxanthin	Protects retina	Enhances absorption	Slows AMD progression	Eggersdorfer and Wyss (2018)
Cardiovascular	Lycopene	Reduces LDL oxidation	Targeted delivery	Lowers CVD risk	Przybylska (2020)
Cognitive	Lutein	Neuroprotective	Blood–brain penetration	Improves memory	Kabir et al. (2022)
Gut health	β‐Carotene	Modulates microbiota	Improves absorption	Reduces inflammation	Silva Meneguelli et al. (2024)
Cancer prevention	Lycopene	Induces apoptosis	Protects degradation	Reduces tumor growth	Zhang et al. (2020)
Liver health	β‐Carotene	Detoxification	Liver‐targeted delivery	Improves enzyme levels	Kang et al. (2021)
Metabolic health	Astaxanthin	Regulates lipids	Enhances solubility	Reduces fat accumulation	Kim et al. (2020)

### 2.7 Cancer Prevention

Due to their anti‐inflammatory and antioxidant properties, which aid in scavenging reactive oxygen species (ROS) and reducing oxidative stress, the principal cause of carcinogenesis. Carotenoids are well known for their cancer‐averting potential (Rowles III and Erdman Jr 2020; Eggersdorfer and Wyss 2018). Lutein, β‐carotene, and lycopene have an extraordinary effect on gene expression, cell signaling pathways, and apoptotic processes that avert the expansion and spread of tumors. For instance, lycopene overwhelms the Wnt/β‐catenin pathway and IGF‐1 while reducing cancer cells' proliferation ability (Saini et al. 2020). Carotenoids comprise β‐carotene, which has been well proven to endorse the natural body detoxification and hence improve the ability to reduce the formation of carcinogenic activation, where it is stimulated by inducing the Phase II detoxification enzymes (Bohn et al. 2021). Epidemiological studies and case studies emphasize the relationship between dietary carotenoid consumption and a reduced risk of cancer. Zare et al. (2021), for example, observed nanodelivery technologies to enhance carotenoid bioavailability and established significant tumor growth suppression in preclinical models. Moreover, through free radical‐scavenging, β‐carotene maintains DNA integrity and decreases the potential for mutagenesis and the onset of cancer (Milani et al. 2017). Clinical trials have also confirmed that lycopene protects against prostate cancer, such that there would be a growth reduction and diminishment of levels of PSA (Eggersdorfer and Wyss 2018). Nonetheless, Black et al. (2020) emphasized the importance of a balanced diet and cautioned against an oversupply of carotenoids, whose potential prooxidative effect is also pertinent. The processes indicate that carotenoids modify numerous pathways related to cancer, comprising the inhibition of angiogenesis, induction of apoptosis in cancerous cells, and inhibition of NF‐κB‐mediated inflammation (Merhan 2017; Young and Lowe 2018). Due to their frequent roles, carotenoids are potent chemopreventive agents for cancer, particularly when combined with other antioxidants for a synergistic effect (Saini et al. 2020).

### 2.8 Neuroprotective Effects

Oxidative stress, neuroinflammation, and apoptosis play an acute role in neurodegenerative diseases such as Alzheimer's disease, Parkinson's disease, and stroke; carotenoids have emerged as a potential neuroprotective agents (Manochkumar et al. 2021; Park et al. 2020). Among the carotenoids, the three possessing significant antioxidant activity are astaxanthin, lutein, and zeaxanthin, which neutralize ROS and reduce lipid peroxidation in neural cells, thus retaining cell membrane integrity (Kabir et al. 2022; Gandla et al. 2023). Through alteration of microglial activity, lycopene has been shown to reduce neuroinflammation and obstruct the aggregation of amyloid‐beta, a hallmark characteristic of Alzheimer's disease (Batool et al. 2022). Such additional neuroprotective effects of carotenoids also comprise case reports and clinical information. For example, lutein supplementation showed improvements in cognition and memory, including retention for elderly people in a comprehensive review conducted by Manochkumar et al. (2021). Such is also stated by Cho et al. (2018), who emphasized that carotenoids do contribute directly to neuroprotection through the alteration of calcium signaling pathways, which are crucial to neuronal existence and to crossing the blood–brain barrier (Rzajew et al. 2020). β‐carotene exhibited important anti‐inflammatory activity in stroke models by plummeting the infarct area and refining functional recovery (Bahonar et al. 2017; Althurwi et al. 2022). Mechanistically, carotenoids act as antiapoptotic drugs by alleviating mitochondrial membranes and averting cytochrome c release, which is a critical step in apoptosis (Park et al. 2020). In addition, they reduce the activation of NF‐κB and inhibit proinflammatory cytokines such as TNF‐α and IL‐6, thus subsidizing their anti‐inflammatory actions (Kabir et al. 2022). Recent studies advise that carotenoids may also moderate neurotransmitter release and neuronal plasticity, which would strengthen their neuroprotective effects (Ahmed et al. 2021). Only a few of the many neuroprotective effects of carotenoids include anti‐inflammatory, antiapoptotic, and antioxidant properties. The growing body of preclinical and clinical indications recommends that the presence of these carotenoids in functional diets and supplements may help control or avoid neurodegenerative diseases (Gandla et al. 2023; Batool et al. 2022).

### 2.9 UV Protection by Carotenoids

Carotenoids such as phytoene, phytofluene, lutein, and β‐carotene are under widespread investigation as UV protectants due to their effective antioxidant properties and aptitude to absorb damaging light wavelengths. These composites eliminate oxidative stress and free radicals, the two main reasons for UV‐induced mutilation (Meléndez‐Martínez et al. 2019; Sandmann 2019). This is what makes carotenoids such as phytoene and phytofluene so superior in that they can decrease photodamage to skin cells. This is because the carotenoids can directly fascinate UV photons. Moreover, carotenoids control intracellular signaling pathways like NF‐κB and MAPK, which are linked with inflammation and apoptosis affected by UV harm (Balić and Mokos 2019).

Additionally, the accessibility of carotenoids influences the increased expression of numerous endogenous antioxidant enzymes, remarkably glutathione peroxidase and superoxide dismutase, on condition that superior defense alongside reactive oxygen species is prompted by UV stimulation (Bohn et al. 2021). Participants of a study evaluating the efficacy of dietary carotenoid supplementation had higher minimal erythema doses (MED), meaning greater resistance to UV‐induced redness, when they consumed a diet rich in β‐carotene (Black et al. 2020). Meléndez‐Martínez et al. (2019) demonstrated that topical solicitations of phytoene and phytofluene‐rich carotenoid extracts dropped visible signs of photodamage, such as collagen degradation and erythema, in UV‐treated animal models. Long‐term cohort studies have established that lesser incidence of skin cancer and photoaging correlate with advanced plasma carotenoid levels (Bungau et al. 2019). Consequently, carotenoids deliver complete photoprotection through the decrease of oxidative stress and strengthening of cellular defense mechanisms.

### 2.10 Improved Respiratory Health

Carotenoids are known to enhance immune responses and protect against oxidative damage in the pulmonary system, thus supporting respiratory health. This function is of specific importance in diseases such as asthma, COPD, and respiratory glitches induced by pollution (Whyand et al. 2018; Saini, Prasad, et al. 2022; Saini, Ranjit, et al. 2022). Carotenoids scavenge ROS and inhibit lipid peroxidation in lung tissues, thereby plummeting inflammation caused by allergens and environmental pollutants. Moreover, they modulate immune cell responses, mainly through enhancing neutrophil and macrophage activity, and suppress proinflammatory cytokines such as TNF‐α and IL‐6 (Mitra et al. 2021; Nabi et al. 2020). Studies have shown that, among others, lycopene and β‐carotene, in particular, inhibit the production of nitric oxide and peroxynitrite, the two key elements liable for inflammation in the airways (Eggersdorfer and Wyss 2018).

In a randomized controlled trial, Whyand et al. (2018) detected that carotenoid supplementation pointedly enhanced pulmonary function tests, such as forced expiratory volume (FEV1), and reduced systemic inflammatory markers in patients with COPD. It has been stated by Bhatt and Patel (2020) that diets rich in carotenoids minimize oxidative stress due to pollution in alveolar cells, thereby plummeting the incidence of respiratory grumbles among the urban population. According to Nabi et al. (2020), those patients who suffer from asthma, taking many carotenoid‐rich diets, have a lesser number of flare‐ups and better lung function as it results in being less hyper‐responsive in their airways. According to population studies, such as those by Mitra et al. (2021), people who consume diets rich in carotenoid content will have significantly lower occurrence rates of respiratory disorders, particularly in areas with high levels of air pollution. Two benefits that carotenoids provide for the deterrence of disorders of the lung include immune‐modulatory and antioxidant properties; this would somehow reduce the negative effects of inflammation and oxidative stress that usually occur in various respiratory diseases. Increasing clinical and epidemiological evidence supports these benefits.

## 3 Encapsulation Techniques for Carotenoids

**FIGURE 2:**
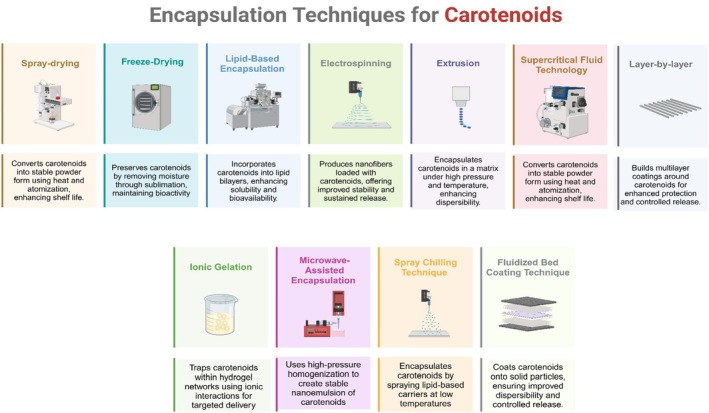
Encapsulation techniques for carotenoid.

Improvements of carotenoids' stability, bioavailability, and functioning may be achieved by encapsulation techniques, which have applications in numerous fields. Such procedures improve the delivery and absorption of carotenoids into the body while providing protection from extraneous elements, such as heat, light, and oxygen that can degrade carotenoids. There have been many developments that differ from each other in their merits regarding the control of release, stability, and potential applications in the food, pharmaceutical, and nutraceutical industries. Some of the most common methods of encapsulating carotenoids are discussed under the following headings, along with an analysis of the underlying concepts, benefits, and practical applications (Figure 2).

### 3.1 Spray Drying

Among the most common techniques for encapsulation of carotenoids is spray drying, based on its efficiency, scalability, and capability to produce fine powder forms. By using heated air, a liquid containing the bioactive component is converted into dry powder. Through a spray nozzle, an emulsion encompassing carotenoids along with suitable wall constituents, like gum arabic or maltodextrin, is atomized into small droplets for spray drying. Hot air, often between 160°C and 200°C, evaporates the water in the droplets after they have adapted to a drying chamber, leaving a solid powder (Eun et al. 2020). Process variables, such as drying time, feed concentration, and input air temperature, are also said to significantly affect carotenoid stability and the efficiency of encapsulation (Pinho et al. 2022; Griep et al. 2024). This process is highly useful for the encapsulation of bioactive compounds extracted from plant sources, like guaraná by‐products, with spray drying since it enhances stability and enables controlled release (Pinho et al. 2022). Moreover, the shelf life of carotenoids is augmented and oxidation is abridged throughout storing due to the encapsulation progression (Chuyen et al. 2019).

### 3.2 Freeze Drying

Freeze drying, often mentioned as lyophilization, is yet another effectual way of encapsulation of carotenoids, mainly for sensitive substances that may break down at a high temperature. A frozen solution of carotenoids is placed in a device and funneled under vacuum with the help of ice, leaving a dry porous structure that holds the biological characteristics of the encapsulated carotenoids. The carotenoids are first mixed with an appropriate excipient like sucrose or trehalose, which might be a cryoprotectant, in order to avert deterioration at freezing. Then, sublimation under reduced pressure is used to expel the frozen mixture (Kandasamy and Naveen 2022). Their molecular structure is not changed since they dry to a steady powder. Since freeze drying is usually carried out at temperatures significantly lower than spray drying, it could be promising for carotenoids (Rezvankhah et al. 2020). Encapsulation of carotenoids gained from oil‐based sources, like gas peel oil, holds superior benefits, as freeze drying would stop the oxidation and degradation of carotenoids (Comunian et al. 2020). Furthermore, the antioxidant properties of carotenoids are preserved with freeze drying (Šeregelj et al. 2021). However, it has limited scalability since its energy and time are more likened to spray drying (Zhu et al. 2021).

### 3.3 Lipid‐Based Encapsulation

The reason these methods of encapsulation by lipid are extremely studied includes refining the stability, bioavailability, and controlled release for lipid‐based encapsulation, particularly the use of liposomes, solid lipid nanoparticles, and nanostructured lipid carriers for the encapsulation of carotenoids. These methods that utilize lipophilic matrices, frequently made of lipids or lipid‐based materials, help in increasing the stability and shelf life of carotenoids, making them more sheltered from environmental degradation like oxidation and light acquaintance (Rehman et al. 2020; Sridhar et al. 2021). Carotenoids are entrapped in the lipid bilayers or in the aqueous interior of liposomes, which are spherical, bilayered vesicles providing protection against degradation by light and oxidation (Assadpour and Jafari 2019). They also augment their bioavailability as they allow hydrophobic substances to liquefy more easily in aqueous conditions (Perera et al. 2023). As per Favas et al. (2024), liposomes have variability in size and charge and the ability to modify their surface for specific targeting and enhanced cell uptake of the encapsulated molecule. Another auspicious encapsulation of the lipid is accomplished using Solid Lipid Nanoparticles (SLNs). It includes the dispersed nature of the carotenoids throughout the lipids. Numerous distinct solid lipids in a watery aqueous solution form nanoparticles that often act as the basis for the formulation of the SLN lipid matrix. SLNs improve the defense mechanisms of carotenoids through reduced heat, light, and oxygen degradation (Tang et al. 2023). As an added advantage, SLNs permit long‐term proclamation, which is indispensable for enhancing carotenoids in functional foods and pharmaceuticals (Barroso et al. 2021).

With the benefits of both SLNs and liquid lipids in a more flexible dual‐phase system, Nanostructured Lipid Carriers is a state‐of‐the‐art lipid‐based system. The combination of solid and liquid lipids in NLCs brings about the following advantages: higher loading capacity, reduced crystallization, and stability of the encapsulated carotenoids (Molteni et al. 2022). According to Dos Santos et al. (2018), it is shown that the stability of carotenoids improves with the increase in stability by NLCs, especially at the time of handling and storage, thereby ideal for their food and nutraceutical application. In contrast, spray drying and freeze drying are conventional methods of encapsulation, which only recently have other studies proven more beneficial for preserving carotenoid stability and enhanced bioavailability than lipid‐based nanostructures (Janiszewska‐Turak 2017; Ledari et al. 2024). Speaking of a couple of lipid‐based carriers, as discussed by Rehman et al. (2020), NLCs and SLNs have their advantage of practically protecting carotenoids from the effects of oxidation while retaining some of their biology (Li et al. 2025). In summary, encapsulation of lipids by liposomes, SLNs, and NLCs offers alternatives for improved carotenoid bioavailability, stability, and controlled release. Such systems are particularly useful within the food and pharmaceutical industries, where the health benefits of bioactive compounds need to be maximized through their protection and delivery improvement (Assadpour and Jafari 2019; Sridhar et al. 2021).

### 3.4 Electrospinning

A new method to encapsulate bioactive compounds like carotenoids within nanofibers is electrospinning. In electrospinning, a polymer solution is spun through an electric field into fine fibers of diameters in the order of nanometers to micrometers (Wen et al. 2017). Because of the very high surface area‐to‐volume ratio of nanofibers, electrospinning is operative at improving the stability, solubility, and bioavailability of carotenoids. This method is very helpful in avoiding oxidative degradation of carotenoids and facilitating their regulated release in culinary and pharmaceutical solicitations (Drosou et al. 2022).

The electrospinning process will expose the polymer solution or melt to an electric field. Then, at the syringe needle tip, this will lead to the formation of a charged jet from the polymer drop (Reksamunandar et al. 2017). With the jet then being stretched and thinned out as it passes through the atmosphere, long nanofibers that can accumulate on a grounded surface are formed. The encapsulated carotenoids include the transporters in the form of nanofibers that protect the bioactive constituents from deterioration. In safeguarding the stability and release kinetics of the encapsulated carotenoids, the assortment of polymers and solvents is important (Xiang et al. 2022).

#### 3.4.1 Types of Electrospinning for Carotenoid Encapsulation

For carotenoid encapsulation, a number of electrospinning varieties has been rummaged, each with exceptional benefits.

##### 3.4.1.1 Coaxial Electrospinning

The use of two concentric nozzles forms a core‐shell fiber structure, in which the outer shell is another polymer with added protection from environmental factors such as light and oxygen, while the carotenoid is embedded within the core (Drosou et al. 2022). Because carotenoids damage upon acquaintance with light and oxygen, coaxial electrospinning is particularly effective in ornamenting their oxidative constancy and photoprotection (Horuz and Belibağlı 2019).

##### 3.4.1.2 Emulsion Electrospinning

The fibers are produced when a carotenoid emulsion is electrospun in a polymer solution, encapsulating emulsion droplets within the polymer matrix, therefore giving the carotenoids a safe and stable abode. This method, however, tends to work rather well with hydrophobic carotenoids when they require an even dispersion of the fiber matrix (Bruni et al. 2020).

##### 3.4.1.3 Blend Electrospinning

A single polymer explanation is applied to liquefy the carotenoid, and that solution is then electrospun into fibers for mixed electrospinning. Despite the nonappearance of an extra outer protective shell to steady the carotenoid, this method is less turbulent and associated with coaxial electrospinning. The efficiency of encapsulation is still pretty high (Reksamunandar et al. 2017).

There are numerous benefits associated with the use of electrospinning to encapsulate carotenoids. Its major advantage is improving oxidative stability. Horuz and Belibağlı (2018) argue that nanofibers might defend carotenoids from some of the degradation elements, such as light, moisture, and oxygen. The regulator of carotenoid release is another main advantage of nanofibers. By changing the composition of the nanofiber matrix, one can regulate the release rate to construct systems suitable for specific applications, such as functional foods or nutraceuticals, where a steady release of carotenoids is predictable (Xiang et al. 2022). Another important advantage of electrospinning is the augmented water solubility of carotenoids. Even though carotenoids are hydrophobic by nature, nanofibers can potentially encapsulate them; thus, they may dissolve in aqueous solutions. This is very valuable in the formulation of water‐based products; therefore, it is crucial for its application (Yildiz et al. 2023). In addition, the electrospinning technique can enclose carotenoids in food‐grade resources; thus, it is appropriate for application in food and beverages (Coelho et al. 2021). Carotenoids were electrospun into strength mats that can be applied in the food industry as functional elements for food products or as covers for food packaging. For example, Bruni et al. (2020) fabricated electrospun fiber mats doped with β‐carotene and reported that they could be applied for food preservation in wrapping. The approach can also enhance the bioavailability of carotenoids in functional foods as encapsulation increases uptake in the digestive tract and protects the chemicals against degradation during digestion (Pinho et al. 2021). Carotenoids associated with electrospun fibers could be utilized for the production of nanostructured drug delivery systems intended to provide medicine at specific site requirements in pharmaceutical applications. It has been documented that Rostamabadi et al. (2020) claim encapsulated carotenoids may have a specific rate of release which can recover efficiency and diminish potential side effects.

### 3.5 Extrusion

Carotenoids constitute naturally occurring pigments belonging to human health in view of their antioxidant properties and acting as precursors for vitamin A. However, these components are highly susceptible to external factors such as heat, light, and oxygen, which severely degrade their functional and nutritional properties. There have been various encapsulation techniques developed to address these issues, and among them, extrusion has arisen as a prevalent method of microencapsulation for carotenoids. Extrusion is the most popular processing method used for food, which extrudes the food into certain forms by forcing the raw material forward through a die under shear force, heat, and pressure. Extrusion enhances the stability and bioavailability of the carotenoids while also protecting the susceptible bioactive molecules when it is applied to the encapsulation process of carotenoids in foods (Pinho et al. 2021). It usually includes integrating carotenoids into carrier ingredients such as proteins, polysaccharides, or lipids to encapsulate the active ingredients and evade their destruction. Bamidele and Emmambux (2021) hold that extrusion creates structures having ideal statement qualities, hydrophobic chemical solubilization, and enhancing sensory properties of the final product. Because it can probably be prepared in substantial quantities, it is inexpensive for food‐based solicitations. This effect of extrusion‐based encapsulation of carotenoids depends on the kind of carrier material used, the concentration of carotenoids, and the extrusion parameters that include temperature, pressure, and screw speed. For instance, it has been shown that the employment of carrier materials such as vegetable lipids, gum arabic, or starch aids the proper stabilization of carotenoids in the processing stage (Pinho et al. 2024). The effectiveness of the process of encapsulation, as well as the defense provided in contradiction of humiliating factors, is contingent on the type of carrier material used. Temperature is also vital during the entire process of extrusion since high temperatures can also reduce the bioactivity of carotenoid pigments. As a result, the alteration of the extrusion conditions is required to uphold the functionalities of the carotenoids and certify efficient encapsulation (Pinho et al. 2021). Studies showed that carotenoid constancy was ensured at relatively lower extrusion temperatures (Bamidele and Emmambux 2021).

It was also used for encapsulation from a wide source of natural extraction such as fruits, vegetables, and microalgae. Such microalgae‐based carotenoids, mainly astaxanthin, have high interest through their strong radical‐scavenging action that is linked with several health‐related benefits (Hamed et al. 2023). It was found through the extrusion process encapsulation with carotenoids extracted from the microalgae that it not only improves bioavailability but protects it from further oxidation. Extrusion will be successful at encapsulating the carotenoid from different origins, including algae and guarana peel. Those carotenoids are generally used in protein‐based matrices or as lipid‐based delivery systems to improve their stability and prevent degradation against processing (Pinho 2022). This new methodology has opened significant avenues for new applications of carotenoids in functional foods as well as dietary supplements.

Encapsulated carotenoids equipped using extrusion technology are obtainable in a wider scope of food products, such as ready‐to‐eat meals, snacks, and functional beverages (Favaro‐Trindade et al. 2020). Encapsulation makes scopes for scheming advanced food products with enhanced nutritional value in addition to authorizing the constancy and bioavailability of carotenoids. As such, Eun et al. (2020) mentioned that improvements in encapsulation via extrusion rely heavily on optimizing the parameters of extrusion, on determining new carriers, and on the integration with other technologies such as spray drying or freeze drying for more progressive constancy and functionality of carotenoids within the food system. These functional ingredients will also upsurge the market as studies will be shown on how encapsulation affects the sensory properties of carotenoid‐enhanced meals (Singh et al. 2023). To put it all together, extrusion signifies a promising substitute for the encapsulation of carotenoids: A chance to protect these bioactive compounds from degradation while enlightening their stability, bioavailability, and conceivable applications in functional foods. Future progressions in extrusion technology will undoubtedly make the applications of carotenoids in the food sector and beyond more imaginable as it leads to even more potent encapsulation techniques (Table 2).

**TABLE 2:** Encapsulation techniques for carotenoids.

Encapsulation technique	Process overview	Advantages	Limitations	Applications	References
Spray drying	Uses hot air to rapidly dry an emulsion into powder form	Cost‐effective, scalable, good retention of carotenoids	Heat exposure may degrade sensitive carotenoids	Functional foods, beverages, supplements	Eun et al. (2020), Janiszewska‐Turak (2017), Corrêa‐Filho et al. (2019)
Freeze drying	Water is removed by sublimation under low temperature and pressure	Protects heat‐sensitive carotenoids, high encapsulation efficiency	Expensive, longer processing time	Pharmaceuticals, high‐value nutraceuticals	Elik et al. (2021), Zhang et al. 2022; Šeregelj et al. (2021)
Lipid‐based encapsulation (Liposomes, SLNs, NLCs)	Carotenoids are enclosed in lipid vesicles or solid lipid carriers	Enhances bioavailability, protects against oxidation	Stability issues, complex formulation process	Drug delivery, fortified foods, functional beverages	Chen et al. (2017), Perera et al. (2023), Sridhar et al. (2021)
Electrospinning	Uses electric force to produce micro‐ and nanofibers for encapsulation	High surface area, controlled release	Limited scalability, process optimization needed	Smart packaging, targeted nutraceuticals	Drosou et al. (2022), Horuz and Belibağlı (2019), Wen et al. (2017)
Extrusion	Encapsulation through pressure and heat into pellets or microbeads	Suitable for large‐scale production, good protection	High temperature may degrade carotenoids	Functional foods, extruded snacks, supplements	Pinho et al. (2024), Bamidele and Emmambux (2021), Favaro‐Trindade et al. (2020)
Supercritical fluid technology	Uses supercritical CO_2_ or other fluids to encapsulate carotenoids, enhancing solubility and bioavailability	Environmentally friendly, solvent‐free, improves stability and bioavailability	High‐pressure equipment required, expensive processing	Pharmaceuticals, functional foods, nutraceuticals	Mihalcea et al. (2017), Klettenhammer et al. (2020), Tirado et al. (2019), Janiszewska‐Turak (2017)
Layer‐by‐layer (LbL) assembly	Coating carotenoid‐loaded particles with alternating polymeric layers for controlled release and protection	Enhances stability, controlled release, prevents oxidation	Complex process, requires precise conditions	Functional foods, nutraceuticals, pharmaceuticals	Lim and Roos (2017), Huang et al. (2024), Pinheiro et al. (2019), Kim, Choi, et al. (2018), Kim, Kim, et al. (2018)
Ionic gelation	Uses polysaccharide‐based matrices (e.g., alginate, chitosan) to form gel networks for encapsulation	Biodegradable, cost‐effective, mild processing conditions	Limited encapsulation efficiency for hydrophobic compounds	Food, pharmaceuticals, nutraceuticals	Otálora et al. (2018), Vakarelova et al. (2023), de Moura et al. (2018), Milivojević et al. (2023)
Microwave‐assisted encapsulation	Uses microwave energy to assist in encapsulation, improving processing speed and efficiency.	Faster processing, enhanced extraction and encapsulation efficiency.	Potential degradation of heat‐sensitive carotenoids.	Functional foods, bioactive compound delivery.	Aldana‐Heredia et al. (2024), İşçimen and Hayta (2021), Saini, Prasad, et al. (2022), Saini, Ranjit, et al. (2022)
Spray chilling	Converts carotenoids into stable, solid microcapsules via rapid cooling of melted lipids	Cost‐effective, protects against oxidation, good for fat‐soluble compounds	Lower encapsulation efficiency than some methods	Food industry, dietary supplements	Eun et al. (2020), de Freitas Santos et al. (2021), Hamed et al. (2023)
Fluidized bed coating	Uses fluidized air to coat carotenoid particles with a protective layer for stability and uniformity	Uniform coating, controlled release, scalable	Requires specialized equipment, may affect particle size	Functional foods, nutraceuticals, pharmaceuticals	Singh et al. (2023), Pinho et al. (2021), Bera et al. (2024)

### 3.6 Supercritical Fluid Technology

Due to their antioxidant properties, there is a wide variety of applications for carotenoids, a family of natural pigments, in the use of food, cosmetics, and pharmaceuticals. Since these bioactive molecules are sensitive to environmental conditions such as heat, light, and oxygen, encapsulation techniques are needed for their protection. One of the most widespread ones is supercritical fluid (SCF) technology, which could encapsulate carotenoids but retain their constancy and bioactivity. Encapsulation events work predominantly well with supercritical fluids and particularly with supercritical CO_2_. These are fluids revealing the physiognomies of both gases and liquids at pressures and temperatures above their harmful pressure and temperature. Bioactive compounds such as carotenoids can be encapsulated and improved very proficiently using SCFs due to their duality, which eases high solubility levels and quick diffusion rates (Klettenhammer et al. 2020). The carotenoids are subjected to a precipitation or encapsulation process exploiting numerous carrier materials following softening through supercritical CO_2_ extraction by SCF expertise. The second benefit of SCF encapsulation is that it can survive under milder environments. Thus, the fragile carotenoids will be improved conserved from this. As noted by Mihalcea et al. (2017), carotenoid bioactivity might be conserved using SCF encapsulation because it results in lower thermal degradation as associated with any other traditional heat‐induced process. Besides, since SCF technology negates the necessity of organic solvents, this technology is far more environmentally friendly than any traditional encapsulation technology (Klettenhammer et al. 2020). Carotenoids from bases such as sea buckthorn and microalgae have been encapsulated using the SCF method—Mihalcea et al. (2017) utilize supercritical CO_2_ to encapsulate carotenoids from sea buckthorn. The constancy of carotenoids encapsulated in whey protein matrices was much better than for nonencapsulated counterparts. This had the consequence of cumulative the bioavailability of carotenoids within meals, with the controlled release permissible by encapsulation (Mihalcea et al. 2017).

Another instance is the employment of supercritical fluid technology for the encapsulation of astaxanthin, a potent pigment resulting from microalgae. Tirado et al. (2019) took a supercritical method to the encapsulation of astaxanthin into ethyl cellulose carriers after an exhaustive continuous emulsion extraction technique. Our process yielded high capability in encapsulation, prolonged release profile, and antioxidant activity, as the health benefit of astaxanthin is hypothetical to be preserved in the application of functional food (Tirado et al. 2019). In the context of carotenoid encapsulation, SCF technology has quite a few compensations over traditional processes. This process can be done at low temperatures to preserve the integrity of heat‐sensitive carotenoids (Mihalcea et al. 2017). In addition, for the full utilization of the announcement and bioavailability of carotenoids, SCF technology also possesses an outstanding resistor over encapsulation competency and particle size (Klettenhammer et al. 2020). Lastly, with the usage of supercritical CO_2_ as a solvent, the process is cleaner because the final product comprises no residues or hazardous solvents. SCF expertise, in addition, allows using any type of carriers for encapsulation. For any kind of carotenoids, either hydrophobic or hydrophilic carrier can be utilized. Thus, this will lead to the probable creation of the standard functional food products and dietary supplements (Janiszewska‐Turak 2017). There are, however, numerous matters that are yet to be talked about despite SCF technology showing much promise in carotenoid encapsulation. The main weakness is that the chief machinery obligatory for processes connecting supercritical fluids entails a very high expense, which may bound the technology's scale. Still, investigation is ongoing and may make this technology more available to large‐scale commercial production by refining SCF trials and reducing equipment costs (Li et al. 2024).

Moreover, though SCF encapsulation has exhibited excellent stability and bioavailability, further research is needed to identify the long‐term stability of encapsulated carotenoids under diverse environmental and storage conditions (Soukoulis and Bohn 2018). Extension of the use of SCF‐encapsulated carotenoids in the food industry would also prerequisite being studied regarding their sensory characteristics and application in diverse food matrices. Incorporating carotenoids using supercritical fluid technology is a feasible, ecofriendly method to encapsulate them and improve their constancy and bioavailability. In contrast, SCF encapsulation offers the possibility to use supercritical CO_2_ as a solvent with mild conditions under which sensitive carotenoid compounds are protected. Challenges in this area remain about equipment cost and scalability, though ongoing developments in SCF technology and its solicitation in carotenoid encapsulation present a rosy future for this technique within the food and nutraceutical industries.

### 3.7 Layer‐By‐Layer (LbL)

Layer‐by‐layer construction is a multipurpose and actual technique for encapsulating carotenoids, which are prone to degradation by factors such as light, oxygen, and heat. With this approach, discontinuous layers of oppositely charged materials are consecutively deposited on a substrate or core material; these are most often biopolymers or nanoparticles. Therefore, a multilayer covering is formed that improves bioavailability, steadies the encapsulated bioactive composites, and allows for controlled release. Because LbL assembly provides precise control over the characteristics of the enclosed system, comprising steadiness, release behavior, and particle size, it is mainly beneficial for encapsulating carotenoids. Suitable biopolymers or suitable nanoparticles that enable stable multilayer structures need to be recognized for the typical approach of using the LbL assembly encapsulation of carotenoids. Lecithin, dextran sulfate, alginate, and chitosan, in particular, are some of the common materials since they can lead to the film generation that happens to be relatively strong, durable, biocompatible, as well as capable of biodegradation. The first step includes revealing the core material, which could be an emulsion or a nanoparticle encumbered with carotenoid, to a polyelectrolyte solution. The early layer of the multilayer structure is shaped when the negatively or positively charged polyelectrolytes adsorb onto the surface of the core material. This process, when repeated with layers of materials having opposite charges, creates a thick and protective layer around the carotenoid core (Wang et al. 2024). The first advantage that can be derived from the LbL assembly is the change in the structure, composition, and thickness of the encircling layers. According to Huang et al. (2024), for a specific carotenoid, the encapsulation system can be adjusted for the required amount of protection from the environmental elements that would otherwise break down carotenoids. Carotenoids can be encapsulated so that their controlled release is permitted by altering the preparation procedure usually preferred for purposes such as nutraceutical and functional foods application (Wang et al. 2023). One of the most astonishing examples of the use of LbL assembly for the encapsulation of carotenoids has been in stabilizing astaxanthin—an antioxidant carotenoid extracted from microalgae and some types of shellfish. Huang et al. combined lecithin, chitosan, and alginate to LbL assemble around astaxanthin. In this case, the chemical encapsulation of astaxanthin also protected the resultant microcapsules from oxidation and breakdown during storage because they were more steady. Song et al. (2022) also fabricated multilayer coatings of chitosan and dextran sulfate by LbL assemblage to encapsulate crocin, a carotenoid extracted from saffron. Because of its greater constancy and bioavailability, encapsulated crocin is especially suitable for utilization in pharmaceutical as well as in functional food products. Extra interfacial structure of LbL was made use of by Lim and Roos (2017) to stable carotenoids in high solvents emulsion during spray drying. Treating the used glass‐forming trehalose, high DE maltodextrin was used on the carotenoids to attempt further encapsulated the carotenoids from decay during the dry process. In addition to improving the stability, the LbL structure improved the solubility and bioavailability of carotenoids, which are two important factors for efficient distribution in food matrices (Lim and Roos 2017). LbL encapsulation technology would be the best option for dispersing and safeguarding carotenoids for numerous reasons. Because of its multilayer assembly, the formulation has outstanding resistance to heat, light, and oxygen factors that have been proven to damage carotenoid pigments (Bera et al. 2024). Due to the consistency of this element, carotenoids increase the shelf life of foodstuffs containing them, thus being better for commercial uses. Controlled release is also allowed by the LbL method in maintaining the bioavailability of carotenoids in humans. This is possible because, as stated by Ge et al. (2024), the quantity and conformation of the layers can be controlled to make the declaration profile distribute a continuous release throughout time. Additionally, LbL technology can encapsulate a wide range of carotenoids, such as hydrophobic and hydrophilic ones, depending on the materials used for the interfacial layers (Pinheiro et al. 2019). Due to its flexibility, it has the ability to encapsulate an extensive diversity of carotenoids from plants, microalgae, and synthetic compounds. Although there is so much promise in the nature of LbL assembly for encapsulation of carotenoids, it still has a lot of challenges. The current LbL technology is among the biggest troubles in scaling up to large‐scale production. Although it is possible to do LbL assembly in a laboratory, the methods and equipment needed to do so are expensive and often hard to use (Bera et al. 2024). More research needs to be conducted to find the best encapsulation conditions for each carotenoid so that this method can be used in industry without losing the efficiency and economy of the method. Future research would most likely focus on the further enhancement of LbL‐encapsulated carotenoid stability by new materials and techniques such as including antioxidant chemicals or the application of more advanced deposition techniques for better coating uniformity (Yan et al. 2019). Moreover, the growth of LbL encapsulation in delivering carotenoids along with other bioactive compounds would be further pushed by the continually increasing demand from consumers for functional foods and nutraceuticals. This increase would likely arise from greater interests in achieving proper control over the release profiles, as well as the overall improvement in the encapsulated products. Layer‐by‐layer assembly is considered one of the most promising methods that can potentially adapt to encasing carotenoids, owing to its great promise in significantly enhancing stability and bioavailability together with controlled release of these biologically important active ingredients. Being customized, it regulates the size and composition of encapsulating layers and prevents further degradation of the encapsulated carotenoids when carefully applied toward delivering them properly within functional food or nutraceuticals. Even though LbL technology has its share of problems concerning scalability, its study and further development will ultimately ensure that the application is enhanced within the pharmaceutical and food sectors.

### 3.8 Ionic Gelation

A widely used approach to encapsulating bioactive agents such as carotenoids involves ionic gelation, involving the formation of gel‐like structures by ionic connections among biopolymers and divalent cations. It is thus highly useful in preventing deterioration and enhancing stability in addition to carotenoid release management. In the preparation of gel beads or microparticles that encapsulate carotenoids inside the beads in a highly effective way, an encapsulation technique makes use of polysaccharides like pectin or alginate along with a crosslinking agent, calcium, or another, according to Naranjo‐Durán et al. (2021). First, it involves the creation of a carotenoid‐loaded emulsion, in which carotenoid is dispersed through an aqueous solution comprising emulsifiers and stabilizers. The mixture is then blended with the biopolymer solution, like sodium alginate, under strictly controlled conditions. Upon introduction into a solution of calcium ions, gelation encapsulates the carotenoids within stable microspheres due to the ionic interaction between alginate and calcium ions (Kurozawa and Hubinger 2017). Before encapsulated carotenoids are delivered as a product, they are washed and dried. This usually involves freeze drying or spray drying. According to Soukoulis and Bohn (2018), the technique offers numerous advantages for the conservation of carotenoids' bioactivity through the reduction of exposure to several factors that can cause depletion, such as heat, light, and oxygen. The resulting solution is mixed with the biopolymer solution, which could include sodium alginate, under controlled conditions. Alginate and calcium ions produce steady microspheres that gel around the carotenoids when unprotected to a calcium ion solution by ionic bonding (Kurozawa and Hubinger 2017). To make the final product, the encapsulated carotenoids are unconcerned from their capsules, separated from their inside organs, and dehydrated typically through freeze drying or spray drying. This technique minimizes the exposure of carotenoids to oxygen, light, and heat, thus offering significant benefits in preserving their bioactivity (Soukoulis and Bohn 2018).

Furthermore, the high encapsulation efficiency of ionic gelation is excellent for carotenoids, which are lengthily rummaged in the food, pharmaceutical, and nutraceutical industries for their antioxidant properties (Rehman et al. 2020). Recent studies have now proven that carotenoid‐incorporated particles can also be stabilized and be bioaccessible if added in combination with other gelling polymers, including chitosan or pectin with alginate (Vakarelova et al. 2023). The ionic gelation process may also change the size, shape, and release characteristics of encapsulated carotenoids when pH, ion concentration, or crosslinking conditions are altered (Shaaban et al. 2024). Due to the aptitude of the ionic gelation method to adapt to an extensive range of encapsulation conditions, it is a very potent and versatile transport and stability technology for carotenoids. Generally, the encapsulation of carotenoids by the ionic gelation method would be a dependable and effective means that would achieve improved stability along with regulated release profiles. It is the best choice for exploiting carotenoids in nutraceuticals and functional foods because it may avert degradation, thereby refining stability and bioavailability (Hamed et al. 2023; Li et al. 2024).

### 3.9 Microwave‐Assisted Encapsulation

Fruits and vegetables include a class of bioactive particles known as carotenoids, which have complicated numerous eyes because of their prospective health assistances and antioxidant possessions. Still, their functionality in food and pharmaceutical applications is controlled by the susceptibility of the composites to the deleterious effects of heat, light, and oxygen (Khalid et al. 2023). Encapsulation is promising in enhancing carotenoid constancy and bioavailability by encapsulating the carotenoids in a protective matrix. Microwave‐assisted encapsulation is a new effective approach in the encapsulation process, providing frequent welfares with regard to processing time, energy efficiency, and encapsulation efficiency. Microwave‐assisted encapsulation, thus, combines the advantages of the traditional encapsulation techniques with extrusion or spray drying while employing microwave radiation to improve the efficiency of the process involved. It provides a homogenous encapsulation of the carotenoids within a carrier material by speeding up the creation of microcapsules through microwave light. One of the primary advantages associated with microwave‐assisted encapsulation is that this process reduces exposure time to hot temperatures as materials are directly heated at the molecular level. Hence, the encapsulation time would be significantly short, and to keep carotenoids stable that are heat‐sensitive, it must be very much essential (Aldana‐Heredia et al. 2024; Pinho et al. 2024). The encapsulation method protects carotenoids during processing and storage by usually using lipid‐based carriers or natural polysaccharides, such as gum arabic (González‐Peña et al. 2023). These carriers create a barrier that averts carotenoids from degrading by obstructing oxygen, light, and heat and upsurges their solubility and bioavailability when ingested (Bamidele and Emmambux 2021). The carrier material is primarily prepared in a form of aqueous solution or emulsion by the microwave‐assisted method. The application of microwave irradiation leads to fast evaporation of the solvent and the encapsulation of the particles. Finally, it has been observed by Pinho et al. (2024) that this technique produces micro‐ or nanoparticles with great carotenoid concentrations and wonderful retention when food is processed or stored. Impressively impressive results are seen to be obtained when other techniques like extrusion become associated with the microwave‐assisted encapsulation process. This technique may bring enhancement to the nutritional profile of food matrices, such as that for snack foods through the incorporation of encapsulated carotenoids (Pinho et al. 2021). Based on these reports, microencapsulated carotenoids possess better properties for heat and oxidation resistance when compared to free forms (Hamed et al. 2023; Soukoulis and Bohn 2018). With the use of microwave‐assisted encapsulation, particle size, surface charge, and the release profile of the final product can be adjusted. The features of Saini, Prasad, et al. (2022) and Saini, Ranjit, et al. (2022) state that such properties determine the bioavailability of encapsulated carotenoids. With microwave‐assisted encapsulation, carotenoids can benefit from improved transport, stability, and bioavailability. This approach has applications both in food and medicine. It is an excellent method for the production of nutraceuticals and functional meals because it saves more energy, processes products more efficiently, and encapsulates the products to a higher extent. Further improvement in the method will lead to a more efficient encapsulation system and thus more uptake of carotenoids in ornamental health goods (González‐Peña et al. 2023; Pinho et al. 2022).

### 3.10 Spray Chilling Technique

In the spray chilling microencapsulation procedure, a liquid is atomized into minor droplets that are then rapidly cooled to solidify the substance inside. This method typically uses a cooling medium, such as cold air, to cool the droplets after they have been sprayed. Because spray cooling has several merits, including improvement in solubility, rate of release, and protection of the bioactive matters from degradation, it has found applications in carotenoids encapsulation (Pinho et al. 2022). Since carotenoids are sensitive to heat, light, and oxygen, this helps them by resisting environmental factors such as heat and light and consequently enhancing their food product stability. To confirm even distribution, spray freezing is carried out on the carotenoid‐rich extracts, which are previously mixed with appropriate carrier ingredients such as gum arabic or maltodextrin. The resulting mixture is then atomized into droplets using a spray nozzle by pumping the mixture into the nozzle. Droplets are instantly cooled by a blast of cold air or liquid nitrogen, solidifying the encapsulated carotenoids. Typically in powder form, the final product is collected and tested for stability, encapsulation efficiency, and carotenoid content (de Freitas Santos et al. 2021). Optimal critical parameters such as carrier‐to‐core ratio, inlet temperature, and atomization pressure achieve high encapsulation competence and steadiness.

### 3.11 Fluidized Bed Coating Technique

Fluidized bed coating is another common method used to encapsulate sensitive bioactive substances such as carotenoids. This technology coats material by suspending small particles in fluidized air flow around the needed. In this process, tiny particles fluidize with a protective layer coated. The coating material can be lipid or polymer‐based. Coating deposition is uniform and controlled nowadays due to fluidized bed technology, protecting the carotenoids from oxygen, light, and heat, thereby stabilizing them (Benelli and Oliveira 2019). This method is useful for industrial usage and ensures that the encapsulation is constant. To make a uniform slurry, fluidized bed coating suspends the high carotenoids extracts in a lipid or polymer that is compatible with the process. Spraying the atomized solution into the fluidized bed chamber with the injection of warm air keeps the particles in motion, and hence, a homogeneous coating is obtained. Controlling the air velocity, humidity, and temperature enhances the process as a whole. The coated particles were then assessed for stability, controlled release, and encapsulation efficacy after drying (Pereira et al. 2021). This technique has gained popularity due to the ability to regulate the thickness of the coating layer with excellent accuracy because it offers superb protection for particularly sensitive carotenoids and applications related to food (Haas et al. 2020).

### 3.12 Ultrasound‐Assisted Encapsulation of Carotenoids

Through the application of sonic cavitation, an innovative process termed ultrasound‐assisted encapsulation enhances carotenoid extraction and encapsulation efficiency. The process facilitates carotenoids to be incorporated into encapsulation systems such as alginate beads, liposomes, or nanoemulsions (Savic Gajic et al. 2021) and their release from plant matrices. Ultrasonic wave‐produced microbubbles implode to produce localized high pressure and temperature, thus disrupting cell membranes and promoting penetration by solvent (Mehta et al. 2022). Improved carotenoid extraction yields, such as for β‐carotene and lutein, from materials such as orange peel, tomato wastes, and microalgae (Cano‐Lamadrid et al. 2025) are the consequence. Homogeneous particle sizes in encapsulating carriers such as calcium alginate beads or liposomes are assisted with ultrasound. By 20%–30% over conventional methods, ultrasonic treatment during encapsulation within alginate beads boosted carotenoid retention, based on Savic Gajic et al. (2021). Ultrasonic waves further enhance carotenoid dispersion in emulsion‐based systems, thus increasing their water solubility and oxidative stability (Chen et al. 2017). Ultrasonic‐assisted encapsulation further aids stabilizing carotenoids in functional meals. For instance, Hamed et al. (2023) observed that in thermal processing and storage conditions, microalgal carotenoids encapsulated in ultrasonic waves were more stable. In chitosan‐TPP systems, Esposto et al. (2022) also proved ultrasonic‐assisted encapsulation increased the bioavailability of carrot‐derived carotenoids.

### 3.13 Hybrid Encapsulation Methods for Carotenoids

By combining two or more methods like ultrasonic with spray drying, solvent evaporation, or ionic gelation, hybrid encapsulation systems facilitate overcoming limitations like low solubility and degradation by capitalizing on the strengths of multiple encapsulation systems to improve the stability and bioavailability of carotenoids. As demonstrated by Savic Gajic et al. (2021), who maximized encapsulation efficiency (EE) up to 85%, one common procedure is ultrasonic‐assisted extraction followed by calcium alginate ionic gelation. Chen et al. (2017) also formulated another method via ultrasonic‐assisted high‐pressure homogenization to get β‐carotene nanoemulsions, thereby enhancing EE by 15%–20% compared to traditional emulsification. Li et al. (2024) also found that by reducing particle size and enhancing homogeneity, coupled solvent evaporation with ultrasonic treatment increased the EE of lutein in polymeric nanoparticles. By protecting carotenoids against oxidation, light, and heat (de Queiroz et al. 2022), such hybrid technologies deliver greater stability alongside increased bioavailability through nanoencapsulation, thus increasing uptake in the gut (Gutiérrez et al. 2013).

In addition, technologies used for targeted and controlled release within food matrices encompass alginate‐chitosan beads (Dos Santos et al. 2018) and TPP‐chitosomes (Esposto et al. 2022). Enhancing carotenoid efficiency, stability, and bioavailability relies significantly on hybrid and ultrasonic encapsulation methods. While ultrasonic waves enhance particle homogeneity and extraction, hybrid methods integrate most strategies for the utmost protection and controlled release, rendering them necessary for nutraceutical and functional food applications to ensure carotenoid stability upon processing and storage.

### 3.14 Nanoencapsulation of Carotenoids

Very susceptible to degradation by light, heat, and oxidation, nanoencapsulation has emerged as a promising method to enhance the stability, bioavailability, and controlled release of carotenoids (Soukoulis and Bohn 2018). Each of the various nanoencapsulation techniques emulsification—, nanoprecipitation, spray drying, and layer‐by‐layer assembly has—special advantages based on the desired application (Gutiérrez et al. 2013). Of these, emulsion‐based technologies are typically applied because they assist hydrophobic carotenoids in becoming more water‐dispersible. Petito et al. (2022), for instance, demonstrated that red bell pepper carotenoids under diverse storage conditions were considerably more stable when nanoemulsions stabilized by diverse encapsulating agents, such as whey protein isolate and gum arabic, were used. In food matrices, Gutiérrez et al. (2013) also observed that high‐pressure homogenization‐formed nanoemulsions enhanced the chemical stability of β‐carotene. Employing biopolymeric nanoparticles, where polymers such as zein, chitosan, and alginate act as encapsulation matrices, is another effective technique. Studying nanoencapsulation of β‐carotene using zein, Mahalakshmi et al. (2020) demonstrated that particle size had a direct influence on release kinetics; smaller‐sized nanoparticles released faster in simulated gastrointestinal conditions. In addition, emphasized by Dos Santos et al. (2018) were polymeric nanoparticles developed using ionic gelation or nanoprecipitation that may provide controlled release and protect carotenoids from environmental stress. Encapsulation of astaxanthin has been successful, thereby boosting its stability and bioavailability, with chitosan–tripolyphosphate (TPP) nanoparticles (Panagiotakopoulos and Nasopoulou 2024).

Another commonly employed technique for carotenoid nanoencapsulation, particularly in the food industry due to its scalability and cost‐effectiveness, is spray drying. Spray‐dried carotenoid powders exhibit improved shelf life and solubility using wall materials like maltodextrin and modified starch, as González‐Peña et al. (2023) described. Also shown to maximize encapsulation stability and efficiency are hybrid methods that integrate multiple techniques, such as ultrasonic‐assisted emulsification followed by spray drying (Amin et al. 2015). The purpose of application dictates the selection of materials and methods to encapsulate; parameters such as particle size, encapsulation efficacy, and release pattern are hence very significant. Advances in recent times in the area of nanoencapsulation have ensured their preservation and bioavailability during processing and storage, thus expanding carotenoids' potential in pharmaceuticals, medicinals, functional foods, and nutraceuticals (Soukoulis and Bohn 2018). Investigation is considering innovative concepts such as solid lipid nanoparticles and nanostructured lipid carriers as it progresses to enhance the delivery and activity of carotenoids in a variety of industrial applications (Dos Santos et al. 2018).

## 4 Optimization of Encapsulation for Different Applications

For developed stability, bioavailability, and controlled release of carotenoids for many applications in the food, pharmaceutical, and cosmetic segments, encapsulation is vital, given that these modern encapsulation techniques are custom made for industry‐specific necessities because they are highly vulnerable to deprivation through light, oxygen, and heat (González‐Peña et al. 2023).

### 4.1 Food Applications

Carotenoids have become very noteworthy in the food industry due to their natural colorant properties as well as other health benefits via their antioxidant potential. However, due to their poor aqueous solubility and sensitivity to process circumstances (Chen et al. 2017; Boonlao et al. 2022), they demand encapsulation techniques such as liposomes, emulsion‐based delivery systems, and nanoencapsulation. Noteworthy research has been carried out on emulsion‐based systems, specifically nanoemulsions, with a view to enhance the dispersibility and chemical stability of β‐carotene. This will consequently enhance β‐carotene's bioaccessibility in dietary matrices (Focsan et al. 2019). It has also been recognized that through protection of carotenoids from oxidation, lipid‐based nanocarriers enhance the intestinal absorption of these substances (Rehman et al. 2020). In food uses, carotenoids are often encapsulated through spray and freeze‐drying processes, which retain their bioactivity while enabling controlled delivery along the digestive tract (Eun et al. 2020). Uniting such drying techniques with maltodextrin or whey protein‐based biopolymer‐based carriers enhances shelf life and functional properties (Sridhar et al. 2021). One of the latest methods to fortify functional meals with carotenoids, expending sustainability and usual biocompatibility, is microalgae‐based encapsulation (Vieira et al. 2020).

### 4.2 Pharmaceutical Applications

The pharmaceutical industry is highly involved in carotenoids due to their potential anticancer, anti‐inflammatory, and antioxidant properties. In medicine delivery, encapsulation technologies have been applied to improve bioavailability and targeted direction. Therapeutic carotenoids can be precisely unconfined and sheltered by lipid‐based nanoparticles, particularly solid lipid nanoparticles (SLNs), and nanostructured lipid carriers (NLCs) (Saini, Prasad, et al. 2022; Zhang et al. 2024; Saini, Ranjit, et al. 2022). Furthermore, the absorption of carotenoid pigments by the body upsurges with supramolecular multiplexes that improve solubility (Focsan et al. 2019). Bioavailability glitches of carotenoids have been overwhelmed using microencapsulation and polymeric nanoparticle techniques, which confirm controlled release and encourage cellular uptake (Rostamabadi et al. 2019). Some recent breakthroughs in encapsulation techniques that involve chitosan and alginate also delivered promising outcomes, including carotenoid distribution and the improvement of gastrointestinal stability in delivery systems (Hamed et al. 2023). Additionally, the applications of encapsulation methods that utilized the microalgae‐derived carrier were also evaluated for medicinal use such as drug targeting treatment and antioxidant therapy (da Silva et al. 2024).

### 4.3 Cosmetic Applications

Carotenoids are expressively practical in cosmetic preparations due to their antiaging, photoprotective, and skin brightening properties. Encapsulating techniques have to be employed to effectively retain effectiveness and shelf life since they have been established to be volatile in formulations. Liposome and nanoemulsion‐based encapsulating technologies are applied in cosmetic formulations to advance skin absorption and constrain oxidative degradation (Li et al. 2024). These were also rummaged through from polymeric microcapsules and cyclodextrin inclusion developments to guarantee the regulated release of carotenoids in cosmetic formulations (Aanniz et al. 2024). This is evident from recent studies that focus on the application of kenaf plant extracts both from leaves and seeds in cosmeceuticals, owing to the encapsulated carotenoids within them that could potentially be involved in skin regeneration and antiaging (Chu et al. 2021). This description additionally suggests that the normal source and extraordinary bioavailability of microalgal carotenoids founded beauty products are the skins that energy them into cosmetics (Hamed et al. 2023).

The growth of encapsulation approaches is important to recover the constancy, bioavailability, and performance of carotenoids in food, medicine, and cosmetic solicitations. For each problem, particular encapsulation processes are obligatory for each corporation. Problems may involve enhanced solubility in food products, prolonged release in cosmetics, or controlled delivery in pharmaceuticals. Nanotechnology, biopolymer‐based encapsulation, and microalgal carriers deliver a foundation for more effective and long‐lasting carotenoids (Table 3).

**TABLE 3:** Optimization of carotenoid encapsulation for food, pharmaceutical and cosmetic applications.

Encapsulation method	Industry application	Key benefits	Limitations	Examples	References
Nanoemulsions	Food, Pharma	Enhanced bioavailability, stability	Requires surfactants, potential oxidation	β‐Carotene, Lycopene	Islam et al. (2023), Chen et al. (2017), Boonlao et al. (2022)
Lipid‐based nanoparticles (SLN, NLC)	Pharma, Cosmetics	Controlled release, high encapsulation efficiency	Expensive, complex production	Lutein, Zeaxanthin	Rostamabadi et al. (2019), Saini, Prasad, et al. (2022), Saini, Ranjit, et al. (2022)
Spray drying	Food, Pharma	Cost‐effective, scalable	Heat‐sensitive compounds may degrade	Astaxanthin, β‐Carotene	Eun et al. (2020), Vieira et al. (2020)
Freeze drying	Food, Pharma	Improved stability, long shelf life	Time‐consuming, costly	Canthaxanthin, β‐Carotene	Eun et al. (2020)
Microencapsulation (Alginate, Chitosan)	Pharma, Cosmetics	Biocompatible, sustained release	May affect release kinetics	Zeaxanthin, Capsanthin	Hamed et al. (2023), da Silva et al. (2024), Afzaal et al. (2022)
Supramolecular complexes	Pharma	Increased solubility, bioavailability	Limited large‐scale use	Astaxanthin, Lycopene	Focsan et al. (2019)
Liposomes	Pharma, Cosmetics	Biocompatible, enhances skin penetration	Prone to leakage, costly	β‐Carotene, Lutein	Li et al. (2024), Aanniz et al. (2024)
Cyclodextrin complexation	Food, Pharma	Improves water solubility	Limited solubilization capacity	Lycopene, Lutein	Li et al. (2024)
Protein‐based encapsulation	Food	Natural carrier, high biocompatibility	Lower protection against oxidation	Astaxanthin, β‐Carotene	Vieira et al. (2020)
Polymeric nanoparticles	Pharma, Cosmetics	Controlled release, bioavailability	Complex formulation	Canthaxanthin, Lutein	Rostamabadi et al. (2019)
Hydrogel‐based encapsulation	Pharma, Cosmetics	Moisture retention, controlled release	Limited structural stability	β‐Carotene, Zeaxanthin	Aanniz et al. (2024)
Microalgae‐based systems	Food, Pharma	Sustainable, natural origin	High production cost	Fucoxanthin, Lutein	Hamed et al. (2023), Vieira et al. (2020)
Solid dispersions	Pharma	Enhanced dissolution	Expensive, not widely used	Lycopene, β‐Carotene	Focsan et al. (2019)
Electrospraying	Pharma	High encapsulation efficiency	Needs optimization for scaling	Zeaxanthin, Astaxanthin	Saini, Prasad, et al. (2022), Saini, Ranjit, et al. (2022)
Spray chilling	Food	Suitable for heat‐sensitive compounds	Limited applications	β‐Carotene, Capsanthin	Eun et al. (2020)
W/O/W emulsions	Food, Pharma	Double‐layer protection, controlled release	Complex formulation	β‐Carotene, Lutein	Rehman et al. (2020)
Nanostructured lipid carriers (NLCs)	Pharma, Cosmetics	Increased carotenoid loading, better absorption	Costly production process	Canthaxanthin, Lutein	Sridhar et al. (2021)
High‐pressure homogenization	Food, Pharma	Increased dispersibility	Requires specialized equipment	β‐Carotene, Lycopene	Rehman et al. (2020)
Coacervation	Food, Cosmetics	Cost‐effective, suitable for hydrophobic compounds	May have low encapsulation efficiency	β‐Carotene, Zeaxanthin	Li et al. (2024)
Encapsulation in biopolymer matrices	Food, Pharma	Sustainable, biocompatible	May affect sensory properties	Lutein, Capsanthin	Vieira et al. (2020), da Silva et al. (2024)

## 5 Challenges and Future Directions in Carotenoid Encapsulation

The main challenges of carotenoid encapsulation are constancy, bioavailability, and scalability. Since they are susceptible to dilapidation by heat, light, and oxygen, carotenoids are less applicable in food and medicine (Soukoulis and Bohn 2018). Though lipid‐based carriers and nanoemulsions have been deliberate to augment stability, it is still challenging to certify a long shelf life (Eun et al. 2020). Bioavailability also constitutes one of the significant issues with carotenoids since they are lipophilic composites insoluble in water. Because of this, there is low absorption into the human body (Boonlao et al. 2022). Though, throughout present times, encapsulation methods such as micelles, liposomes, and cyclodextrins have shown promise in ornamental bioaccessibility despite inherent problems in commercial manufacture (Focsan et al. 2019). One other disadvantage is that the encapsulating technology costs. This mainly relates to industrial purposes. High energy input and the specialized equipment in advanced encapsulation techniques like spray drying, freeze drying, and nanoparticle‐based technologies raise the cost of production (Rostamabadi et al. 2019). Vieira et al. (2020) reported that the commercialization of new encapsulating resources is restricted by restrictions on their permission. Despite such limitations, there are several promising tendencies that influence how carotenoid encapsulation will progress in the future. The constancy and bioavailability of carotenoids have been boosted by advancements in encapsulating techniques. These two new approaches involving nanostructured lipid carriers and microencapsulation involving biopolymers are promising research areas intended to improve preservation and controlled carotenoid delivery (Hamed et al. 2023).

Further scientific studies on plants and ocean source carotenoids may provide avenues for greater environmental sustainability and in‐use applications for carotenoids (Chuyen and Eun 2017). Improving bioavailability by new delivery techniques is the other big trend. Recent years have witnessed surfactant‐based nanoemulsions and protein‐based carriers to enhance the absorption of carotenoids in the GI tract (Chen et al. 2017). Some of the examples of bioactive mediators that were shown to increase bioefficacy through co‐encapsulation are polyphenols and omega‐3 fatty acids (Saini, Prasad, et al. 2022; Saini, Ranjit, et al. 2022). Encapsulated carotenoids must still remain acceptable to the consumer before any of these would be acceptable in food and in nutraceutical products. Clean label encapsulation systems are being advanced using natural carriers such as pectin, chitosan, and alginate, an effort to market these to health‐conscious customers (González‐Peña et al. 2023). Growing requests for diet supplements and personalized nutrition also give way to encapsulated carotenoids because of progressions in 3D food printing and functional foods (Li et al. 2024). In summary, carotenoid encapsulation is yet to be relatively easy, although new investigations and technical progresses now open the gates to more well‐organized, affordable, and user‐friendly alternatives. These would necessitate that innovative encapsulation methods and sustainable carotenoid sources be implemented to overcome present constraints and raise their uses in food, medicine, and cosmetics (Zabot et al. 2022).

## 6 Conclusion and Future Perspectives

Bioactive substances of high strength, carotenoids have anti‐inflammatory, immune moderating, and antioxidant properties. They are very appreciated in human nutrition and health due to their preventive functions against chronic diseases like cancer, neurodegeneration, and cardiovascular disorders. Carotenoids have, though, been prevented from widespread usage in drugs and functional foods because of their low bioavailability and inherent volatility. Because of their potential to advance stability, controlled release, and the higher absorption rate of carotenoids, encapsulation has been a growing effort in addressing these concerns. A wide array of methodologies, such as lipid‐based encapsulation, spray drying, and supercritical fluid technologies, have shown promise in making carotenoids more bioavailable and protecting them from environmental degradation. Some encapsulation methods, including lipid‐based carriers, are better suited to increase solubility and absorption, while others, such as electrospinning and layer‐by‐layer assembly, improve targeted administration and controlled release based on a comparison of these approaches. Despite this progress in the encapsulation of carotenoids, problems persist relating to consumer acceptance, scalability, and cost‐effectiveness. Future research must focus on improving these techniques to balance cost‐effectiveness with efficacy, as well as ensuring compliance with the regulations for safe application in the food, pharmaceutical, and cosmetic industries. To unlock the full potential of carotenoids as applied to human health, interdisciplinary approaches and the incorporation of novel technology will be fundamental.

Carotenoid encapsulation is a fast‐developing field, and the investigation is accompanied to recover industrial scalability and bioavailability. In the future, progresses are predictable to include nanotechnology, biopolymer‐based delivery systems, and advanced green encapsulating techniques to create more effective and sustainable carotenoid formulations. The use of bio‐based polymers and natural emulsifiers can enhance the biocompatibility and functional enactment of encapsulated carotenoids, thereby making them more suitable for clean label and ecofriendly product formulations. Additionally, clever delivery methods like stimuli‐responsive nanoparticles that release carotenoids rendering to specific physiological conditions would further improve their therapeutic application. This will drive the implementation of carotenoid‐enriched products for cosmeceuticals, targeted medication distribution, and customized nutrition. Improving hybrid encapsulation systems comprising numerous techniques that improve stability, solubility, and bioavailability is an additional interesting direction of future investigation. Lipid‐based carriers combined with electrospinning or supercritical fluid technology can advance the controlled release and defensive properties of carotenoids. In addition, the predictability of the interactions between carotenoids and encapsulating material becomes probable using the development of computational modeling and machine learning, thus allowing the creation of more operative encapsulation systems. Long‐term safety assessments and regulatory endorsement procedures should also be engrossed on by future investigation to ensure the commercial viability of carotenoid‐based encapsulated products. Carotenoids will promise to recover human health when these problems are tackled and creative strategies are exploited, leading to their broader usage in medications, nutraceuticals, and functional foods.

## Author Contributions

**Muhammad Tayyab Arshad:** writing – original draft (equal). **Sammra Maqsood:** writing – review and editing (equal). **Ali Ikram:** validation (equal). **Ammar Ahmad Khan:** supervision (equal). **Awais Raza:** formal analysis (equal). **Aneeq Ahmad:** conceptualization (equal). **Kodjo Théodore Gnedeka:** visualization (equal).

## Disclosure

The authors have nothing to report.

## Ethics Statement

The authors have nothing to report.

## Consent

The authors have nothing to report.

## Conflicts of Interest

The authors declare no conflicts of interest.

## Data Availability

The data supporting this study's findings are available from the corresponding author upon reasonable request.
